# Mesenchymal Stem Cells-Induced Trophoblast Invasion Is Reduced in Patients with a Previous History of Preeclampsia

**DOI:** 10.3390/ijms23169071

**Published:** 2022-08-13

**Authors:** Reyna Peñailillo, Stephanie Acuña-Gallardo, Felipe García, Lara J. Monteiro, Gino Nardocci, Mahesh A. Choolani, Matthew W. Kemp, Roberto Romero, Sebastián E. Illanes

**Affiliations:** 1 Laboratory of Reproductive Biology, Center for Biomedical Research and Innovation (CIIB), Universidad de los Andes, Santiago 7620001, Chile; 2 Faculty of Medicine, Universidad de los Andes, Santiago 7620001, Chile; 3 IMPACT, Center of Interventional Medicine for Precision and Advanced Cellular Therapy, Santiago 7620001, Chile; 4 Molecular Biology and Bioinformatics Lab, Program in Molecular Biology and Bioinformatics, Center for Biomedical Research and Innovation (CIIB), Universidad de los Andes, Santiago 7620001, Chile; 5 Department of Obstetrics and Gynaecology, Yong Loo Lin School of Medicine, National University of Singapore, Singapore 119228, Singapore; 6 Division of Obstetrics and Gynaecology, The University of Western Australia, Crawley, WA 6009, Australia; 7 Perinatology Research Branch, Division of Obstetrics and Maternal-Fetal Medicine, Division of Intramural Research, *Eunice Kennedy Shriver* National Institute of Child Health and Human Development, National Institutes of Health, United States Department of Health and Human Services, Bethesda, MD 20892, and Detroit, MI 48201, USA; 8 Department of Obstetrics and Gynecology, University of Michigan, Ann Arbor, MI 48109, USA; 9 Department of Epidemiology and Biostatistics, Michigan State University, East Lansing, MI 48824, USA; 10 Center for Molecular Medicine and Genetics, Wayne State University, Detroit, MI 48201, USA; 11 Detroit Medical Center, Detroit, MI 48201, USA

**Keywords:** preeclampsia, MenSCs, trophoblast invasion

## Abstract

Endometrial stromal cells play an important role in reproductive success, especially in implantation and placentation. Although Mesenchymal stem cells (MSCs) have been studied to assess decidualization disorders in preeclampsia (PE), their role during trophoblast invasion remains unclear. This study aims to determine: (i) whether MSCs isolated from menstrual fluid (MenSCs) from nulliparous, multiparous, and women with a previous history of preeclampsia exhibited different patterns of proliferation and migration and (ii) whether reproductive history (i.e., prior pregnancy or prior history of PE) was able to produce changes in MenSCs, thus altering trophoblast invasion capacity. MenSCs were collected from nulliparous and multiparous women without a history of PE and from non-pregnant women with a history of PE. Proliferation and migration assays were performed on MenSCs with sulforhodamine B and transwell assays, respectively. Trophoblast invasion was analyzed by culturing HTR-8/SVneo trophospheres on a matrigel overlying MenSCs for 72 h at 5% O_2_, simulating a 3D implantation model. A previous history of pregnancy or PE did not impact the proliferative capacity or migratory behavior of MenSCs. Following exposure to physiological endometrial conditions, MenSCs demonstrated upregulated expression of *IGFBP-1* and *LIF* mRNA, decidualization and window of implantation markers, respectively. The mRNA expression of *VIM*, *NANOG*, and *SOX2* was upregulated upon trophosphere formation. Relative to co-culture with multiparous MenSCs, co-culture with PE-MenSCs was associated with reduced trophoblast invasion. The findings of this study suggest a potential role for communication between maternal MenSCs and invading trophoblast cells during the implantation process that could be implicated in the etiology of PE.

## 1. Introduction

Preeclampsia (PE) is a pregnancy-specific disorder characterized by new-onset hypertension and proteinuria after 20 weeks of gestation [1]. It affects 2–8% of all pregnancies and is associated with an increased risk of maternal and fetal morbidity and mortality [2]. Although the precise etiology of PE remains unclear, it is now widely accepted that its pathophysiological process involves deficient trophoblast invasion of the maternal decidua and impaired remodeling of the maternal spiral arteries during the first trimester of pregnancy [3,4]. For successful implantation and placentation, the interaction between decidual stromal cells and extravillous trophoblast (EVT) cells is crucial. Specifically, trophoblast invasion relies on communication between the blastocyst and the maternal decidua [5].

The endometrium is highly dynamic and undergoes cyclical regeneration, differentiation, and shedding during the menstrual cycle. In humans, decidualization occurs during the mid-secretory phase of the cycle, begins around the spiral arteries, and is independent of the presence of the conceptus [6]. During this process, regulated by estradiol and progesterone, cells acquire a secretory phenotype that discharges specific products such as prolactin and insulin-like growth factor binding protein 1 (IGFBP1) [7]. Decidualization helps regulate embryo implantation and, subsequently, cytotrophoblast interaction with the uterus, making this process an essential component of establishing the maternal–fetal interface during normal pregnancy [8]. Failed decidualization has been an important contributor of altered cytotrophoblast invasion in human endometrial stromal cells from women with a previous pregnancy complicated by severe PE [9].

Placentation requires the invasion of fetal-derived EVT cells into the maternal uterine spiral arteries [10]. EVTs, that differentiate from cytotrophoblast cells, lose some epithelial phenotypes at the villous tips and acquire additional mesenchymal phenotypes, improving their migration and invasion capacity. EVT invasion involves the degradation and remodeling of the extracellular matrix, which is achieved mainly by matrix metalloproteinases (MMPs), such as MMP-2 and MMP-9 [11], and alters the abundances of adhesion-associated molecules such as E-cadherin and vimentin [12,13]. Alteration in MMP expression and activity could cause uterine and vascular dysfunction, contributing to the pathogenesis of PE [14].

Endometrial cells have been demonstrated to play a central role in normal and abnormal early pregnancy development. Mesenchymal stem cells (MSCs) are pluripotent progenitor cells with a self-renewing capacity and potential ability to differentiate. MSCs of endometrial origin have been identified and characterized from human endometrial tissue and menstrual fluid [15,16]. The properties of MSCs isolated from menstrual fluid (MenSCs) have demonstrated improved angiogenic properties, including vascular endothelial growth factor (VEGF) secretion, in comparison to MSCs isolated from the bone marrow [17]. We previously studied the angiogenic properties of MenSCs obtained from patients with a history of PE (PE-MenSCs), further demonstrating less endoglin and VEGF expression as well as less VEGF secretion but higher expression of interleukin (IL)-6 compared to MenSCs obtained from women with a previous normal pregnancy [18]. These results suggested that PE-MenSCs had reduced angiogenic capacity and were more proinflammatory than those of MenSCs from women with a previous normal pregnancy [18].

In this study, we aim to: (i) characterize the proliferative capacity and migratory behavior of MenSCs isolated from women with or without a history of PE (nulliparous and multiparous women); and to (ii) determine the capacity of MenSCs to induce trophoblast invasion by utilizing a blastocyst-like structure in a 3D in vitro model that simulated communication between endometrial and trophoblast cells.

## 2. Results

### 2.1. Characteristics of the Donors

The demographic characteristics of the nulliparous, multiparous, and PE donors are presented in Table 1. Nulliparous donors were younger than multiparous and PE women (adjusted *p* values = 0.021 and *p* = 0.008, respectively); however, there was no difference between multiparous and PE women (adjusted *p* value > 0.99). Body mass index and gravidity did not differ between groups. Preeclamptic women presented with significantly lower gestational ages and newborn weights in their last pregnancy compared to multiparous women (control group).

### 2.2. Proliferation and Migration of MenSCs

**Figure 1:**
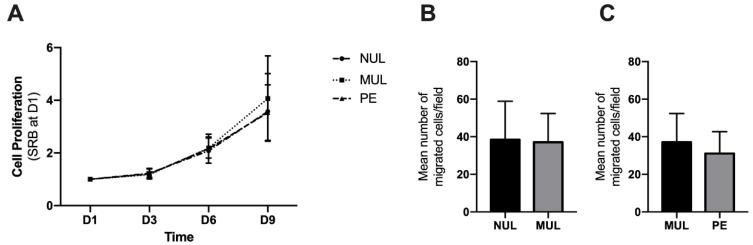
Proliferation and migration of MenSCs derived from nulliparous (NUL), multiparous (MUL) and PE women. (**A**) MenSCs were seeded for SRB assays to quantify cell proliferation at day 3, day 6, and day 9. Results are presented as mean values and SDs of eight MenSCs per group. (**B**) NUL and MUL MenSCs migration assays. (**C**) MUL and PE MenSCs trans-migration assays. Results are presented as mean values and SDs of six MenSCs per group. Statistical analyses were performed with Mann–Whitney tests. MenSCs, mesenchymal stem cells derived from the menstrual fluid; PE, preeclampsia; SD, standard deviation; SRB, sulforhodamine B.

To assess functional properties of MenSCs, the proliferative potential and migratory behavior of cells obtained from the 3 different groups were compared. The proliferation rate was similar in nulliparous (NUL), multiparous (MUL), and preeclamptic (PE) women (Figure 1A) from day 1 to day 9. The migratory behavior of MenSCs isolated from multiparous women did not differ from nulliparous MenSCs (*p* = 0.393, Figure 1B) or from MenSCs isolated from women with a history of PE (*p* > 0.999, Figure 1C).

### 2.3. Estradiol and Progesterone Treatment Increase the Expression of LIF and IGFBP1 in MenSCs

**Figure 2:**
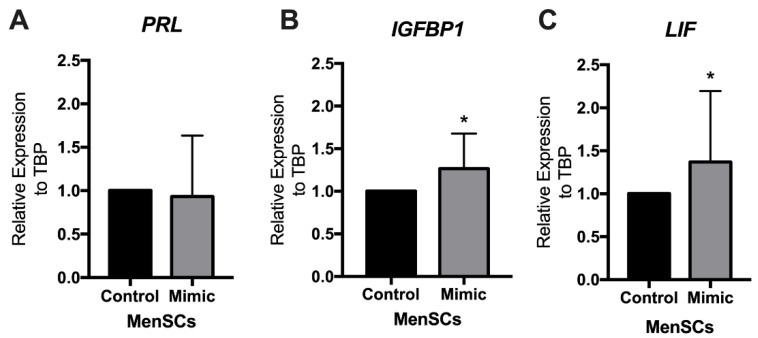
Characterization of MenSCs after endometrial physiological conditions (Mimic). Gene expression of decidualization markers: (**A**) Prolactin (PRL) and (**B**) insulin-like growth factor binding protein 1 (IGFBP1) were detected in MenSCs. Window of implantation marker (**C**) leukemia inhibitory factor (LIF) was detected in MenSCs after hormonal treatment. Results are expressed as mean and SD of nine MenSCs per group. * *p* < 0.05. Statistical analyses were performed by using the Wilcoxon matched-pairs signed-rank test. MenSCs, mesenchymal stem cells derived from the menstrual fluid; SD, standard deviation.

To characterize the effect of estradiol and progesterone treatment on MenSCs cultured in 5% O_2_ (endometrial physiological conditions, defined as Mimic), the gene expression of decidualization and the window of implantation markers were evaluated in all MenSCs. We determined the expression of insulin-like growth factor binding protein 1 (IGFBP1) and prolactin (PRL) as decidualization markers. *IGFBP1* demonstrated a significant increase (*p* = 0.02, Figure 2B) compared to cells without hormonal treatment (control). MenSCs also demonstrated an increase in leukemia inhibitory factor (LIF) (*p* = 0.04, Figure 2C), the window of implantation marker after treatment with the endometrial hormonal mimic, suggesting that the exposure of MSCs of menstrual origin to estrogen for 24 h and then to estrogen plus progesterone for another 24 h at levels of oxygen present in the endometrium can simulate endometrial conditions that precede implantation.

### 2.4. Characterization of Trophospheres from HTR-8/SVneo Cells Origin

**Figure 3:**
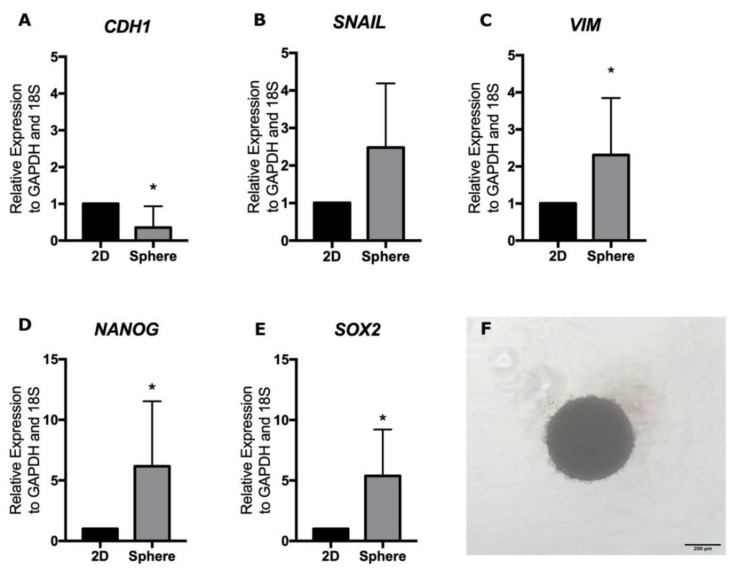
Characterization of trophospheres after 72 h culture compared to 2D adherent culture. mRNA expression of *CDH1* (**A**), *SNAIL* (**B**), *VIM* (**C**), *NANOG* (**D**) and *SOX2* (**E**) in trophospheres and 2D cultures was analyzed by qRT-PCR and normalized to *GADPH* and *18S* housekeeping genes. (**F**) Tropho-sphere following 72 h of culture. Results are expressed as mean and SD of seven trophospheres and HTR-8/SVneo monolayer cultures per group. * *p* < 0.05. Statistical analyses were performed by using the Wilcoxon matched-pairs signed-rank test. SD, standard deviation.

Relative to adherent 2D cultures, trophospheres had a significant decrease in mRNA expression of the epithelial marker *CDH1* (*p* = 0.046, Figure 3A), an increase in mRNA expression of the mesenchymal gene *VIM* (*p* = 0.03, Figure 3C) and the stemness markers *NANOG* and *SOX2* (*p* = 0.015 both, Figure 3D,E) after 72 h in culture. There was no difference in the expression of mesenchymal *SNAIL* transcript levels in the trophospheres compared to the adherent cells (*p* = 0.21) (Figure 3B). These findings suggest spheroid formation and culture are associated with the induction of epithelial-mesenchymal transition (EMT) and that the formed spheres are able to express higher levels of stemness genes.

### 2.5. The Invasive Potential of Trophospheres Is Reduced When Co-Cultured with PE-MenSCs

**Figure 4:**
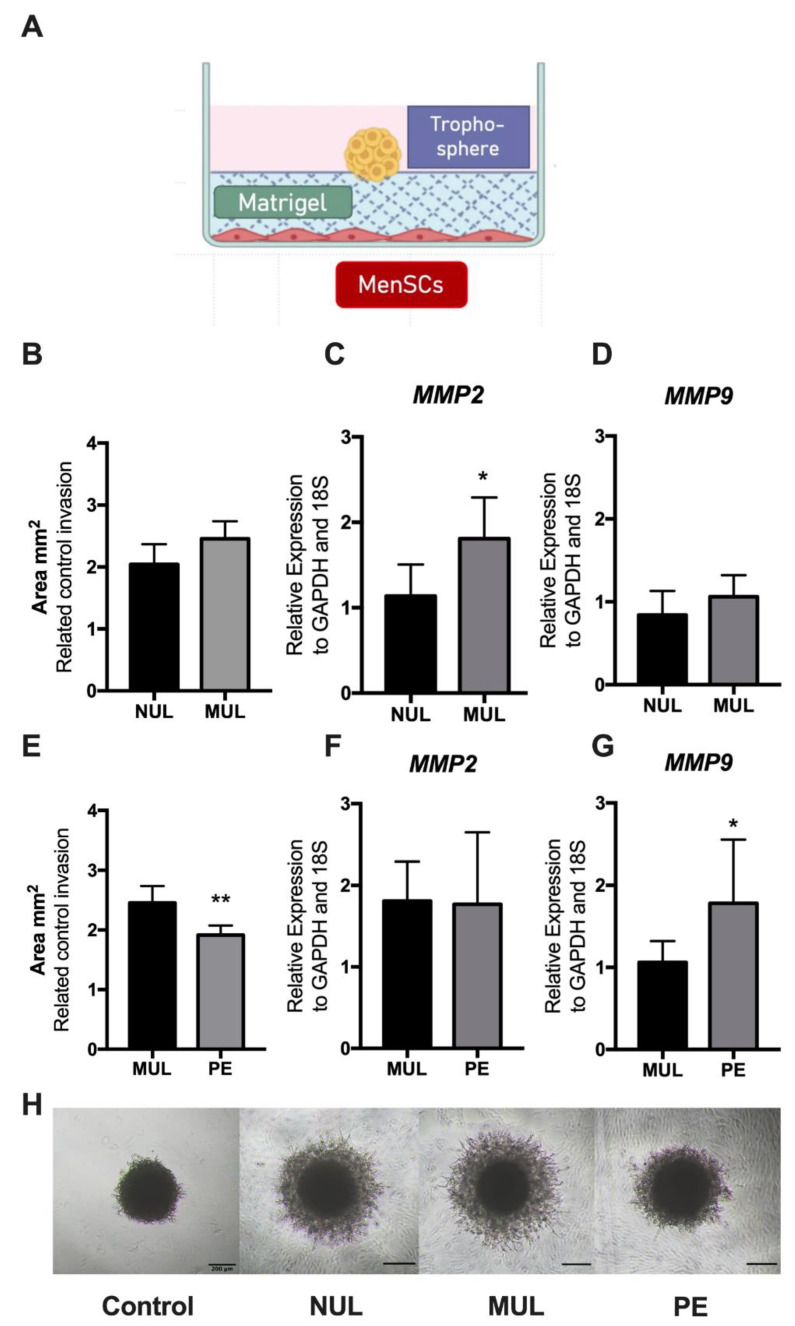
In vitro 3D model of trophoblast invasion. (**A**) Schematic depicts the components of the model: MenSCs on the bottom of the plate as a monolayer of endometrial cells. Matrigel mimics the extracellular matrix, and trophospheres mimic the trophectoderm of a blastocyst that is transferred onto the Matrigel. (**B**,**E**) Invasion area of trophospheres co-cultured with MenSCs of nulliparous, multiparous, and PE women. Each area is expressed as relative to control invasion without MenSCs. Results are the means and SD of six MenSCs per group. (**C**,**F**) *MMP2* and (**D**,**G**) *MMP9* mRNA expression in trophospheres after invasion assay compared to the control invasion trophospheres. * *p* < 0.05, ** *p* < 0.01. Statistical analysis was performed by using the Mann–Whitney U test. (**H**) Representative images of trophospheres invasion after 72 h of co-culture with MenSCs. MenSCs, mesenchymal stem cells derived from the menstrual fluid, SD, standard deviation.

Trophoblast invasion involves an active process of migration through various layers of endometrial tissues, including the extracellular matrix and stroma. To elucidate the communication between endometrial stem cells and trophoblasts during the invasion process, we developed a 3D in vitro invasion model by using MenSCs, Matrigel, and trophospheres (Figure 4A). After 72 h of co-culture, we observed the formation of projections that penetrated the Matrigel in a radial orientation. Using phase-contrast images captured with an inverted microscope, we measured the area of invasion and compared it to the area of invasion measured in 3D cultures lacking MenSCs (Figure 4H). First, we analyzed the effect of gravidity on trophoblast invasion. The results demonstrated that when trophospheres are co-cultured with MUL-MenSCs there was a modest but non-significant difference in the degree of invasion compared to NUL-MenSCs (*p* = 0.064, Figure 4B). These trophospheres had an increased expression of matrix metalloproteinase 2 (*MMP2*; *p* = 0.036, Figure 4C). Secondly, we compared whether the PE history of donors can influence trophoblast invasion capacity induced by MenSCs. Indeed, when trophospheres were cultured with MenSCs from women with a history of PE (PE-MenSCs), they had a reduced area of invasion compared to MUL-MenSCs (*p* = 0.004, Figure 4E). Trophospheres co-cultured with PE-MenSCs showed an increased expression of matrix metalloproteinase 9 (*MMP9*) after invasion (*p* = 0.036, Figure 4G) but no difference in *MMP2*.

## 3. Discussion

MenSCs are characterized by their capabilities of self-renewal and differentiation under standard conditions [19]. They are the focus of a growing amount of interest due to their clinical potential in the study of pregnancy complications, given the minimally invasive manner in which they may be obtained. Data published previously suggested that MenSCs retain phenotypic variation postpartum that may be associated with, and perhaps account for the abnormal implantation process observed in PE [18]. Moreover, human decidual natural killer cells also possess a trained memory after the first pregnancy, which includes expression of receptors that interact with EVTs and growth factors promoting an efficient placentation in subsequent pregnancies [20,21]. This pregnancy-imprinted memory could be a part of the reason why a history of PE could impact the capacity to induce trophoblast invasion by MenSCs. In this study, troposphere co-culture with PE-MenSCs was associated with decreased trophoblast invasion capacity compared to the control (MUL-MenSCs). This impaired invasion capacity was not associated with changes detectable in the proliferation and migration capacity of the same cells. However, deficient trophoblast invasion during placentation in combination with maternal conditions are accepted as a cause of endothelial dysfunction, inflammatory alteration, and the appearance of symptoms of PE [22].

The EMT-related molecules are linked to the migration and invasion capacity of the trophoblast. This process includes the reduction of epithelial and the increase in mesenchymal markers in trophoblast cells. Our model of trophospheres demonstrated the induction of EMT as decreasing *CDH1* expression and as increasing *VIM*, *NANOG* and *SOX2* compared to the 2D monolayer culture of the same cells. These results have been reported previously [23,24] and suggest that spheroids have the capacity to drive EVTs, as we observed in our 3D invasion model.

Trophoblast invasion is regulated by several factors, including MMPs, the proteases that degrade different components of the extracellular matrix, and are expressed by utero-placental interphases such as trophoblast, endometrial stromal cells, and natural killers cells [25]. MMPs play a major role in trophoblast invasion into the uterine wall to create an optimum environment for embryonic development. Studies have demonstrated that MMP-2 and MMP-9 may be implicated in the early and late stages of PE due to their role in vasodilation, placentation, and uterine expansion during normal pregnancy [14]. Herein, however, we demonstrated that despite the reduced trophoblast invasion capacity in the presence of PE-MenSCs, the expression of *MMP2* in trophospheres did not exhibit differences after the 72-h invasion compared to the control group (MUL-MenSCs), while *MMP9* demonstrated an increased expression. The altered expression of MMPs have also been reported in preeclamptic women. Higher levels of MMP-2 and lower levels of MMP-9 in maternal serum have been associated with early- and late-onset severe PE [26], and the urinary MMP-2 concentration at 12 and 16 weeks of gestation was reported as increased in women who developed PE later during pregnancy [27].

Trophoblast cell invasion is regulated by signaling events, autocrine and paracrine stimuli, specific protein recognition, and immunological tolerance [28]. Considering our control of invasion without MenSCs, communication between MenSCs and trophoblast cells during the 3D invasion model results appears necessary to induce trophosphere invasion. Therefore, we propose that MenSCs may stimulate trophosphere invasion through the release of exosomes [29]. Studies have demonstrated that exosomes secreted by MenSCs served as a convincing new type of cell-free treatment [30,31]. Exosomes contain microRNA/lncRNA and adhesion molecules as well as small vesicles with secreted proteins, which mediate cellular signaling pathways [32]. The content of exosomes secreted by MenSCs in relation to the invasion capacity remains poorly characterized. Analysis of the MenSCs-derived exosomes could help to explain the changes in the invasion capacity of trophoblast cells.

Given that the human uterus is exposed to varying hormonal profiles throughout the menstrual cycle [33], MenSCs were cultured with estradiol and progesterone to simulate the window of implantation environment in vitro. During the window of implantation, the blastocyst can attach to the endometrial epithelial cells and invade the endometrial stroma and vasculature [34]. The receptive endometrium is characterized by the appearance of pinopodes and the increased expression of the leukemia inhibitor factor (LIF). LIF regulates trophoblast cell adhesion, and it might be important for embryo invasion and placental development. Our results demonstrated an increase in *LIF* expression in MenSCs after treatment with estradiol and progesterone, simulating the signaling milieu present in the uterus. The extent of trophoblast invasion relies on communication between the placenta and maternal decidua. Studies have demonstrated increased decidualization markers such as IGFBP1 and prolactin in endometrial stromal cells after hormonal treatment [35,36]. Moreover, endometrial stromal cells of women with a history of PE failed to decidualize in vitro after hormonal treatment [9]. Herein, we only observed increased of *IGFBP1* expression in MenSCs after hormonal treatment, suggesting that the hormone concentration used in this study might be too modest to induce decidualization.

## 4. Materials and Methods

### 4.1. Isolation and Culture of MenSCs

Menstrual fluid was self-collected by consenting donors following informed consent according to a protocol reviewed and approved by the ethical scientific committee of Universidad de los Andes. Samples of MenSCs were obtained from three groups of study participants: nulliparous women, multiparous women, and women with a history of PE. All donors confirmed that they had not used hormonal contraceptives for at least three months, and a clinical/gynecological history was obtained for each donor.

Overnight menstrual fluid was collected in a silicone menstrual cup within the first 48 h of menstruation and transferred into a 50 mL tube containing 10 mL 1× phosphate-buffered saline (PBS) and the following supplements: 0.25 mg/mL amphotericin B, penicillin 100 IU, streptomycin 100 mg/mL, and 2 mM ethylenediaminetetraacetic acid (EDTA) (all Gibco, Thermo Fisher Scientific, Waltham, MA, USA). MenSCs were isolated from the mononuclear cell fraction with Ficoll^®^Paque Plus (GE Healthcare, Piscataway, NJ, USA) density gradient by centrifugation at 400× *g* for 30 min at room temperature, according to the manufacturer’s instructions. Mononuclear cells were recovered from the interface between the plasma and Ficoll^®^Paque Plus, then washed twice with PBS to remove the platelets, Ficoll^®^Paque Plus, and plasma. Mononuclear cells were cultured in Dulbecco’s Modified Eagle Medium (DMEM) with high glucose (Mediatech Inc, Manassas, VA, USA) supplemented with 1% penicillin/streptomycin (P/S), 2.5 µg/mL amphotericin B, 2 mM glutamine, and 15% fetal bovine serum (FBS) (all Gibco, Thermo Fisher Scientific) in a humidified environment at 37 °C and 5% CO_2_ to obtain adherent cells. All experiments were performed by using MenSCs at early passages (P) P3 to P7.

### 4.2. Proliferation Assay: Sulforhodamine Assay

MenSCs were cultured at 1000 cells/cm^2^ in 24-well plates (Falcon, Corning, NY, USA) in supplemented DMEM (10% FBS, 1% penicillin-streptomycin, 1% glutamine). Cell proliferation and viability were determined at days 3, 6, and 9 by using a sulforhodamine B (SRB) assay (BioVision, Milpitas, CA, USA). The SRB assay is based on the ability of the protein dye SRB to bind basic amino acid residues of fixed cells. Quantification was performed by spectrophotometric quantification (absorbance to 492 nm) on a Tecan Sunrise Reader, 96-well Microplate Reader, according to the manufacturer’s instructions.

### 4.3. Migration Assay

MenSCs were suspended in 400 µL of DMEM with reduced serum (0.5% FBS) and seeded in a millicell insert (pore 8 µm, 12 mm, Millipore, Billerica, MA, USA) with 25,000 cells/insert. Inserts were placed on 500 µL of complete media (10% FBS) in 48-well plates. Migration capacity was evaluated at 18 h. Briefly, the insert was washed with 1× PBS, fixed with cold methanol for 2 min, and stained with 0.5% crystal violet (Winkler, Santiago, Chile). The cells inside the inserts were scraped with cotton swabs moistened with 1× PBS to ensure only migrated cells were analyzed. Five fields were captured for each insert at 40× objective magnification before and after scraping under an inverted microscope (Primo Vert, Zeiss, Jena, Germany), using the AxioCam ERc5s camera (Zeiss). Images were analyzed with AxioVision analysis software (Zeiss). The percentage of migrated cells was calculated as follows: number of cells after/before scraping × 100 (average of the 5 fields). The experiments were performed in duplicate.

### 4.4. Hormonal Treatment

MenSCs were cultured under an endometrial hormonal milieu, as described previously [18]. Briefly, 3000 MenSCs/well were cultured in a 96-well plate with DMEM phenol red free media (Mediatech Inc.), supplemented with 10% charcoal-stripped FBS, 1% penicillin-streptomycin for 24 h. The following day, medium was replaced with fresh media supplemented with 17β-estradiol (E2; 213 pg/mL) and cultured at 5% O_2_ for another 24 h. The day after, MenSCs were exposed to 17β-estradiol and progesterone (E2; 146 pg/mL, P4; 11 ng/mL) and cultured at 37 °C for another 24 h in a humidified atmosphere hypoxia chamber with 5% O_2_ and -5% CO_2_. Hormone-treated MenSCs were used in real-time quantitative reverse transcription PCR (qRT-PCR) analysis or 3D invasion assays.

### 4.5. Trophoblast Sphere Formation

Trophoblast sphere formation was described previously in 2019 [19], which we used with some modifications. An adherent first-trimester trophoblast cell line, HTR-8/SVneo (HTR-8) was purchased from the American Type Culture Collection (CRL-3271; Lot #70016636, ATCC, Manassas, VA, USA). Cells were maintained in RPMI-1640 medium (GE Healthcare, Piscataway, NJ, USA), 10% heat-inactivated FBS (Gibco, Thermo Fisher Scientific), and 1% P/S (Gibco, Thermo Fisher Scientific) at 37 °C in a humidified incubator with 5% CO_2_. For trophoblast sphere formation, HTR-8 cells were harvested and resuspended in complete media. The 2 × 10^4^ HTR-8 cells were suspended in 200 μL and placed into each well of an ultra-low attachment 96-well plate. Following centrifugation at 300× *g* for 5 min, cells were incubated for 72 h at 37 °C in a humidified atmosphere hypoxia chamber with 5% O_2_ and 5% CO_2_. The trophospheres were washed with 1× PBS and used for characterization by qRT-PCR or for 3D invasion assays.

### 4.6. 3D Invasion Assay

This model was first described in 2019 [19], which we used with some modifications. MenSCs treated under endometrial hormonal milieu in 96-well plates were used for 3D invasion assays. Matrigel Growth Factor Reduced and Phenol Red-free (Corning Life Sciences, Union City, CA, USA) was mixed with DMEM containing 10% charcoal-stripped FBS and 1% penicillin-streptomycin 1:1 and added to the treated MenSCs in the 96-well plates. The plate was then incubated for 30 min at 37 °C to allow the Matrigel to solidify. A single sphere of trophoblast cells was subsequently placed on each well onto the Matrigel, and 150 μL of DMEM, 10% charcoal-stripped FBS, and 1% P/S were added to embed the sphere. Trophosphere invasion was evaluated after 72 h. Phase contrast images were captured by the contrast microscope Olympus CKX41 and Axiocam 208 color (Zeiss). The invasion level (area) was quantified by using ImageJ software, and trophospheres were collected for qRT-PCR assay. Eight trophospheres were required for each condition.

### 4.7. RNA Isolation and qRT-PCR

Total RNA was extracted from MenSCs and trophospheres by using TRIzol Reagent (Invitrogen Corporation, San Diego, CA, USA), according to the manufacturer’s protocol. RNA amount and quality were evaluated on the NanoDrop 2000 spectrophotometer (Thermo Fisher Scientific) at 260/280 with all samples having values between 1.8 and 2.0. Before cDNA synthesis, total RNA was treated with DNase I (Invitrogen Corporation). One microgram of RNA was used for reverse transcription using SuperScript II (Invitrogen Corporation), according to the manufacturer’s instructions. Determination of gene expression was carried out using Brilliant III SYBR Green qPCR Master Mix (Agilent Technologies, Santa Clara, CA, USA), according to the manufacturer’s instructions, and amplified on a qRT-PCR Stratagene Mx3000P System (Agilent Technologies). GAPDH and 18S were used as housekeeping genes for the normalization of trophospheres and TBP was used for MenSCs. The real time PCR was set at 95 °C for 10 min for enzyme activation, followed by 40 cycles of denaturation, primer annealing, and extension consisting of 95 °C for 15 s, 60 °C for 15 s, and 72 °C for 15 s, respectively. All samples were run in duplicate. After the PCR runs, a dissociation curve was generated to confirm the absence of nonspecific amplification. The expression was quantified using the 2^−ΔΔCT^ method. Primers details are provided in Table 2. Reaction specificity was confirmed using dissociation curves, no-template, and no-RT controls for each target.

### 4.8. Statistical Analysis

Graphs were made and statistical analyses were performed with GraphPad Prism Version 7.0 software (Graphpad, San Diego, CA, USA). Statistical significance was set at *p* < 0.05 for all analyses. Data normality was tested by the Shapiro–Wilk test. For parametric data (characteristics of donors) we applied one-way ANOVA with a Bonferroni post-test and a Student’s *t*-test and for nonparametric data (functional assays and qRT-PCR), Kruskal–Wallis, Dunn’s multiple comparison, and Mann–Whitney U tests were applied.

## 5. Conclusions

In summary, we demonstrated that PE-MenSCs exposed to trophospheres in a 3D co-culture model was associated with a decrease in the invasive capacity of trophoblasts in vitro, a characteristic that could be associated with the pathogenesis of PE.

## Figures and Tables

**Table 1:** Characteristics of controls and preeclampsia donors.

Characteristics	Nulliparous	Multiparous	Preeclampsia	*p* Value
	(n = 10)	(n = 10)	(n = 9)	
Age (years)	27.6 ± 4.5	34 ± 4.6	35.0 ± 5.3	0.005
BMI (Kg/m^2^)	23.2 ± 3.4	22.2 ± 3.2	24.8 ± 4.7	0.319
Gravidity	0	3.1 ± 1.3	2.1 ± 1.1	0.086
Gestational age at last delivery	-	37.7 ± 1.1	33.3 ± 5.9	0.045
Newborn weight	-	3240 ± 388.2	2100 ± 1014.8	0.004

BMI, body mass index. Statistical analyses were performed with one-way ANOVA with Bonferroni post hoc test for the variables age and BMI (between the 3 groups), meanwhile for gravidity, gestational age at last delivery and newborn weight, Student’s *t*-test was performed (MUL and PE groups).

**Table 2:** Primer sequence and annealing temperatures (AT) for qRT-PCR.

Gene	Sequence	AT °C
18S Fw	GCCGCTAGAGGTGAAATTCTTGGA	60
18S Rv	ATCGCCGGTCGGCATCGTTTAT	
GAPDH Fw	GTCAGGGTCTCTCTCTTCCT	60
GAPDH Rv	GCTCTCCTCTGACTTGAACA	
TBP Fw	CAC GAA CCA CGG CAC TGA T	62
TBP Rv	GTT GGT GGG TGA GCA CAA GG	
SOX2 Fw	AGC TAC AGC ATG ATG CAG GA	60
SOX2 Rv	GGT CAT GGA GTT GTA ATG CA	
NANOG Fw	CTG ATT CTT CCA CCA GTC CC	60
NANOG Rv	AGG TCT TCA CCT GTT TGT AG	
SNAIL Fw	CCC CAA TCG GAA GCC TAA CT	62
SNAIL Rv	GCT GGA AGG TAA ACT CTG GAT TAG	
IGFBP1 Fw	GAA GGA GCC CTC CCG AAT AG	62
IGFBP1 Rv	CCA TTC CAA GGG TAG ACG CA	
CDH1 Fw	CTGCCAATCCCGATGAAATTG	60
CDH1 Rv	TCCTTCATAGTCAAACACGAGC	
VIM Fw	AGTCCACTGAGTACCGGAGAC	62
VIM Rv	CATTTCACGCATCTGGCGTTC	
LIF Fw	GTTTCCTCCAAGGCCCTCT	60
LIF Rv	TGTTTCCAGTGCAGAACCAA	

## Data Availability

Not applicable.

## References

[1] Brown M.A., Magee L.A., Kenny L.C., Karumanchi S.A., McCarthy F.P., Saito S., Hall D.R., Warren C.E., Adoyi G., Ishaku S. (2018). Hypertensive disorders of pregnancy: ISSHP classification, diagnosis, and management recommendations for international practice. Hypertension.

[2] Steegers E.A.P., Von Dadelszen P., Duvekot J.J., Pijnenborg R. (2010). Pre-eclampsia. Lancet.

[3] Albrecht E.D., Pepe G.J. (2020). Regulation of Uterine Spiral Artery Remodeling: A Review. Reprod. Sci..

[4] Pollheimer J., Vondra S., Baltayeva J., Beristain A.G., Knöfler M. (2018). Regulation of Placental Extravillous Trophoblasts by the Maternal Uterine Environment. Front. Immunol..

[5] James-Allan L.B., Whitley G.S., Leslie K., Wallace A.E., Cartwright J.E. (2018). Decidual cell regulation of trophoblast is altered in pregnancies at risk of pre-eclampsia. J. Mol. Endocrinol..

[6] Ng S.W., Norwitz G.A., Pavlicev M., Tilburgs T., Simón C., Norwitz E.R. (2020). Endometrial decidualization: The primary driver of pregnancy health. Int. J. Mol. Sci..

[7] Liu H., Huang X., Mor G., Liao A. (2020). Epigenetic modifications working in the decidualization and endometrial receptivity. Cell. Mol. Life Sci..

[8] Gellersen B., Reimann K., Samalecos A., Aupers S., Bamberger A.M. (2010). Invasiveness of human endometrial stromal cells is promoted by decidualization and by trophoblast-derived signals. Hum. Reprod..

[9] Garrido-Gomez T., Dominguez F., Quiñonero A., Diaz-Gimeno P., Kapidzic M., Gormley M., Ona K., Padilla-Iserte P., McMaster M., Genbacev O. (2017). Defective decidualization during and after severe preeclampsia reveals a possible maternal contribution to the etiology. Proc. Natl. Acad. Sci. USA.

[10] Pijnenborg R., Vercruysse L., Hanssens M. (2006). The Uterine Spiral Arteries In Human Pregnancy: Facts and Controversies. Placenta.

[11] Chen J., Khalil R.A. (2017). Matrix Metalloproteinases in Normal Pregnancy and Preeclampsia. Progress in Molecular Biology and Translational Science.

[12] Turco M.Y., Moffett A. (2019). Development of the human placenta. Development.

[13] Knöfler M. (2010). Critical growth factors and signalling pathways controlling human trophoblast invasion. Int. J. Dev. Biol..

[14] Nikolov A., Popovski N. (2021). Role of gelatinases mmp-2 and mmp-9 in healthy and complicated pregnancy and their future potential as preeclampsia biomarkers. Diagnostics.

[15] Gargett C.E., Schwab K.E., Zillwood R.M., Nguyen H.P.T., Wu D. (2009). Isolation and Culture of Epithelial Progenitors and Mesenchymal Stem Cells from Human Endometrium. Biol. Reprod..

[16] Meng X., Ichim T.E., Zhong J., Rogers A., Yin Z., Jackson J., Wang H., Ge W., Bogin V., Chan K.W. (2007). Endometrial regenerative cells: A novel stem cell population. J. Transl. Med..

[17] Alcayaga-Miranda F., Cuenca J., Luz-Crawford P., Aguila-Díaz C., Fernandez A., Figueroa F.E., Khoury M. (2015). Characterization of menstrual stem cells: Angiogenic effect, migration and hematopoietic stem cell support in comparison with bone marrow mesenchymal stem cells. Stem Cell Res. Ther..

[18] Varas-Godoy M., Acuña-Gallardo S., Venegas-Duarte S., Hill C., Caceres-Verschae A., Realini O., Monteiro L.J., Zavala G., Khoury M., Romero R. (2019). Angiogenic Properties of Menstrual Stem Cells Are Impaired in Women with a History of Preeclampsia. Stem Cells Int..

[19] Patel A.N., Park E., Kuzman M., Benetti F., Silva F.J., Allickson J.G. (2008). Multipotent menstrual blood stromal stem cells: Isolation, characterization, and differentiation. Cell Transplant..

[20] Staff A.C., Fjeldstad H.E., Fosheim I.K., Moe K., Turowski G., Johnsen G.M., Alnaes-Katjavivi P., Sugulle M. (2022). Failure of physiological transformation and spiral artery atherosis: Their roles in preeclampsia. Am. J. Obstet. Gynecol..

[21] Gamliel M., Goldman-Wohl D., Isaacson B., Gur C., Stein N., Yamin R., Berger M., Grunewald M., Keshet E., Rais Y. (2018). Trained Memory of Human Uterine NK Cells Enhances Their Function in Subsequent Pregnancies. Immunity.

[22] Martinez-Fierro M.L., Hernández-Delgadillo G.P., Flores-Morales V., Cardenas-Vargas E., Mercado-Reyes M., Rodriguez-Sanchez I.P., Delgado-Enciso I., Galván-Tejada C.E., Galván-Tejada J.I., Celaya-Padilla J.M. (2018). Current model systems for the study of preeclampsia. Exp. Biol. Med..

[23] Wong M.K., Wahed M., Shawky S.A., Dvorkin-Gheva A., Raha S. (2019). Transcriptomic and functional analyses of 3D placental extravillous trophoblast spheroids. Sci. Rep..

[24] Zhu J.-Y., Pang Z.-J., Yu Y.-H. (2012). Regulation of trophoblast invasion: The role of matrix metalloproteinases. Rev. Obstet. Gynecol..

[25] You Y., Stelzl P., Zhang Y., Porter J., Liu H., Liao A.H., Aldo P.B., Mor G. (2019). Novel 3D in vitro models to evaluate trophoblast migration and invasion. Am. J. Reprod. Immunol..

[26] Laskowska M. (2017). Altered Maternal Serum Matrix Metalloproteinases MMP-2, MMP-3, MMP-9, and MMP-13 in Severe Early- and Late-Onset Preeclampsia. Biomed Res. Int..

[27] Martinez-Fierro M.L., Perez-Favila A., Garza-Veloz I., Espinoza-Juarez M.A., Avila-Carrasco L., Delgado-Enciso I., Ortiz-Castro Y., Cardenas-Vargas E., Cid-Baez M.A., Ramirez-Santoyo R.M. (2018). Matrix metalloproteinase multiplex screening identifies increased MMP-2 urine concentrations in women predicted to develop preeclampsia. Biomarkers.

[28] Staun-Ram E., Shalev E. (2005). Human trophoblast function during the implantation process. Reprod. Biol. Endocrinol..

[29] Adam S., Elfeky O., Kinhal V., Dutta S., Lai A., Jayabalan N., Nuzhat Z., Palma C., Rice G.E., Salomon C. (2017). Review: Fetal-maternal communication via extracellular vesicles—Implications for complications of pregnancies. Placenta.

[30] Rosenberger L., Ezquer M., Lillo-Vera F., Pedraza P.L., Ortúzar M.I., González P.L., Figueroa-Valdés A.I., Cuenca J., Ezquer F., Khoury M. (2019). Stem cell exosomes inhibit angiogenesis and tumor growth of oral squamous cell carcinoma. Sci. Rep..

[31] Dalirfardouei R., Jamialahmadi K., Jafarian A.H., Mahdipour E. (2019). Promising effects of exosomes isolated from menstrual blood-derived mesenchymal stem cell on wound-healing process in diabetic mouse model. J. Tissue Eng. Regen. Med..

[32] Chen L., Qu J., Xiang C. (2019). The multi-functional roles of menstrual blood-derived stem cells in regenerative medicine. Stem Cell Res. Ther..

[33] Liu S.-f., Wang Z.-x., Yuan Y.-e., Bing S.-m., Zhang B.-z., Wu J.-z., Wu Y.-e., Peng X.-y. (1986). Hormone changes during the menstrual cycle of Chinese women. Reproduction.

[34] Ochoa-Bernal M.A., Fazleabas A.T. (2020). Physiologic events of embryo implantation and decidualization in human and non-human primates. Int. J. Mol. Sci..

[35] Skliutė G., Baušytė R., Borutinskaitė V., Valiulienė G., Kaupinis A., Valius M., Ramašauskaitė D., Navakauskienė R. (2021). Menstrual blood-derived endometrial stem cells’ impact for the treatment perspective of female infertility. Int. J. Mol. Sci..

[36] Logan P.C., Ponnampalam A.P., Steiner M., Mitchell M.D. (2013). Effect of cyclic amp and estrogen/progesterone on the transcription of dna methyltransferases during the decidualization of human endometrial stromal cells. Mol. Hum. Reprod..

